# Gender equality in academic psychiatry in the UK in 2019

**DOI:** 10.1192/bjb.2020.116

**Published:** 2021-06

**Authors:** Sukriti Dhingra, Helen Killaspy, Sarah Dowling

**Affiliations:** 1 Division of Psychiatry, University College London, UK

**Keywords:** Gender equality, academic psychiatry, women, gender balance, United Kingdom

## Abstract

**Aims and method:**

To investigate whether gender balance in academic psychiatry in the UK has improved since a 2005 initiative to encourage career progression for female academics in UK universities. We surveyed the gender of academic psychiatrists across the UK and compared our findings with our previous 2003 London-wide survey and with the Royal College of Psychiatrists’ 2001 workforce census.

**Results:**

The percentage of women in academic psychiatry posts in the UK more than doubled, from 20% in 2001 to 40% in 2019, with increases at senior lecturer (from 25 to 50%), reader/associate professor (from 29 to 48%) and professor level (from 11 to 21%). Outside London, men occupy 72% of all posts and 89% of professorial posts. Within London, men occupy 45% of all posts and 74% of professorial posts.

**Clinical implications:**

The representation of women in academic psychiatry has improved but men continue to dominate at professorial level. Gender equality appears worse outside London. The situation is exacerbated by the diminishing availability of posts across the UK.

The percentage of female medical students in the UK in 1963 (the first year that data were reported) was 29%. It had increased to around 40% by 1980 and, since the mid-1990s, it has been consistently greater than 50%.^1^ In 2018–2019, 59% of medical and dental students were female.^2^ However, NHS Digital reported that women made up only 45% of qualified doctors in the UK in 2018 and 64% of consultant posts were held by men.^3^ This suggests that women are more likely to leave medicine or fail to progress to consultant grade than their male counterparts. Gender balance also varies between medical specialties: in psychiatry women held 45% of substantive consultant posts in 2019,^4^ reflecting a gradual increase from the 34% reported in the Royal College of Psychiatrists’ workforce survey in 2004.

Gender balance is worse in clinical academia. A survey published in 2018 by the Medical Schools Council identified 3465 clinical academic posts in the UK,^5^ comprising approximately 2% of the NHS medical workforce.^6^ One-third (31%) of these posts were in London. Most (41%) were in medicine, followed by surgery (9.1%), psychiatry (7.3%) and general practice (7.2%). Men held 72% of all clinical academic posts. Gender imbalance increased with academic seniority, with women occupying 41% of lecturer grade, 34% of senior lecturer/reader grade and 18% of professor grade posts.^5^ In 2003, we conducted a survey of academic psychiatrists employed in substantive posts in London universities and found that only 24% were women, comprising 62% of lecturers, 25% of senior lecturers, 29% of readers and 11% of professors.^7^

Since 2005, higher education institutions in the UK have been encouraged to participate in the Athena Scientific Women's Academic Network (SWAN) charter,^8^ which promotes good practice in addressing inequalities in career progression in fields that have tended to have poorer gender balance – science, technology, engineering, mathematics and medicine (‘STEMM’ subjects). The programme has been incentivised by awarding academic departments, institutions and universities three grades of charter mark (bronze, silver and gold), with the suggestion that only those that achieve silver will be eligible to apply for certain national research funding streams.

The UK Royal College of Psychiatrists’ workforce census of 2019 reported that women held 29% of clinical academic posts in psychiatry.^4^ However, these data were limited by possible double counting of clinical and clinical academic posts, missing data and lack of breakdown by academic grade. We therefore contacted all UK universities known to undertake research in psychiatry to request information about the gender balance of their substantive academic psychiatrists. We aimed to investigate the situation within and outside London universities and to compare our data with our previous London-wide survey results from 2003 and with data from the Royal College of Psychiatrists’ 2001 UK-wide workforce census published in our original survey^7^ to investigate whether, in the context of initiatives such as Athena SWAN, there had been any improvement in gender balance in academic psychiatry.

## Method

We contacted the relevant heads of departments of all 15 UK universities known to employ psychiatrists in substantive academic or clinical academic posts (Imperial College London, King's College London, Queen Mary University London, University College London, the Universities of Bangor, Cambridge, Cardiff, Edinburgh, Liverpool, Manchester, Nottingham, Oxford, Swansea and Warwick, and Queen's University Belfast). We requested data on the number and gender of academic psychiatrists by grade (professor; associate professor/reader; senior lecturer; lecturer; research fellow). No other data were requested, no individually identifiable data were gathered and data were collated across institutions; therefore no ethical approval was required. Departments that did not respond to the first email received two further reminders.

### Analysis

Data were collated using IBM SPSS version 25.0 for Windows. We present descriptive statistics (frequencies and percentages) on the number and gender of academic psychiatrists in the UK and within and outside London. Chi-squared tests were conducted to compare the percentage of women by grade within and outside London, and the change in percentage of women by grade across the UK using the Royal College of Psychiatrists’ workforce census data from 2001, and within London using the data from our previous survey of London academic psychiatrists conducted in 2003.^7^

## Results

We received responses from 12 of the 15 (80%) universities, including all those in London. The gender of academic psychiatrists by grade is shown in Table 1. Overall, 49% of posts were held by women. Although there was equal gender balance at senior lecturer and reader/associate professor level, men occupied 79% of professorial posts.

**Table 1:** Gender balance among UK academic psychiatrists, 2019

Academic grade	Female, *n* (%)	Male, *n* (%)	Total, *n*
Research fellow	60 (70)	26 (30)	86
Lecturer	34 (64)	19 (36)	53
Senior lecturer	36 (50)	36 (50)	72
Reader/associate professor	11 (48)	12 (52)	23
Professor	21 (21)	79 (79)	100
Total	162 (49)	172 (51)	334

**Fig. 1:**
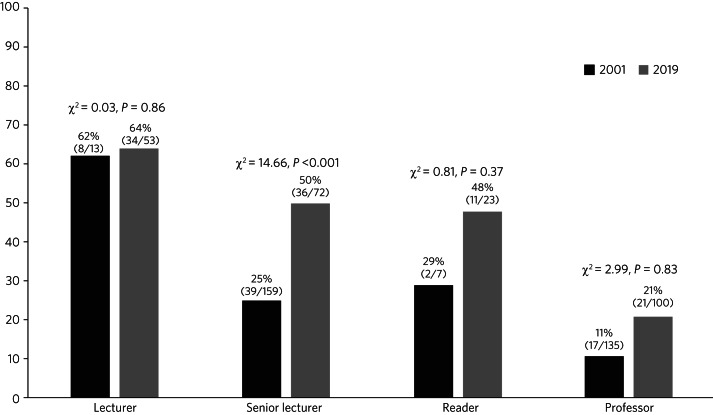
Percentage of UK female academic psychiatrists, 2001 and 2019.

Figure 1 shows the gender balance by grade of UK academic psychiatrists in 2001 and 2019. Research fellow posts are not included as data on these were not reported in 2001. The percentage of female academic psychiatrists has increased from 20 to 40% overall, with the largest increase seen at the level of senior lecturer (from 25 to 50%), a statistically significant increase. Of note, the total number of posts has fallen from 366 to 248 since 2001.

Table 2 shows the gender of academic psychiatrists by grade in universities inside and outside London, including research fellows. Around three-quarters of all UK posts (252/334) were in London. Women held the majority of more junior posts (research fellow and lecturer) within London, whereas the reverse was true outside London (although the total number of these posts outside London was small). These differences in junior posts within and outside the capital were statistically significant. There was equal gender balance within and outside London at senior lecturer grade, but most professorial posts were held by men (89% outside London, 74% within London). Women occupied 63% of reader/associate professor posts outside London and 40% within London, but, again, the number of these posts was relatively small and thus we need to be cautious in interpreting this difference. There were no statistically significant differences in the percentage of women at these higher grades within and outside London.

**Table 2:** Gender balance among academic psychiatrists within and outside London, 2019

	London			Outside London				
Academic grade	Female, *n* (%)	Male, *n* (%)	Total, *n*	Female, *n* (%)	Male, *n* (%)	Total, *n*	Chi-squared	*P*
Research fellow	57 (76)	18 (24)	75	3 (28)	8 (72)	11	10.79	0.001
Lecturer	32 (74)	11 (26)	43	2 (20)	8 (80)	10	10.45	0.001
Senior lecturer	27 (50)	27 (50)	54	9 (50)	9 (50)	18	0	>1
Reader/associate professor	6 (40)	9 (60)	15	5 (63)	3 (37)	8	1.06	0.303
Professor	17 (26)	48 (74)	65	4 (11)	31 (89)	35	3.18	0.074
Total	139 (55)	113 (45)	252	23 (28)	59 (72)	82	18.20	<0.001

**Fig. 2:**
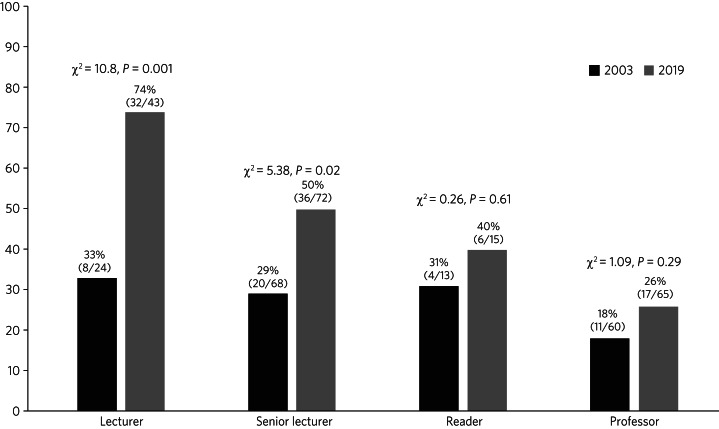
Percentage of female academic psychiatrists, London universities, 2003 and 2019.

Figure 2 shows the gender balance by grade within London universities in 2003 and 2019. Research fellow posts are not included as data on these were not gathered in 2003. The percentage of academic psychiatrist posts in London occupied by men fell from 74% in 2003 to 54% in 2019, with increases in the percentage of women at every grade: a 41% increase at lecturer level, 21% at senior lecturer, 9% at reader/associate professor, and 8% at professor level. However, only the increases in the percentage of women at lecturer and senior lecturer level were statistically significant.

## Discussion

We found 49% of all clinical academic posts in psychiatry to be occupied by women, compared with the 29% reported in the Royal College of Psychiatrists’ 2019 census.^4^ The disparity may be explained by the limitations of the census noted previously (double counting and missing data). Although we did not achieve 100% response from universities outside London, given that three-quarters of posts were within London, this finding appears robust as well as encouraging. Our results also compare favourably with the figure of 28% reported by the Medical Schools Council for all medical specialties.^5^

Our data suggest that gender equality in academic psychiatry across the UK has improved since 2001, with increases in the percentage of women at all grades and a statistically significant increase at senior lecturer level, where women now hold 50% of posts. This is in keeping with the gradual increase in women achieving substantive consultant posts (an equivalent grade to senior lecturer) in psychiatry over a similar period.^4^ However, there has been a comparatively small rise of only 10% in women at professor grade, with four-fifths of these posts occupied by men. This is particularly disappointing when comparing these figures with other medical specialties, where women still only comprise one-third of senior lecturer and readers/associate professors and 18% of professors.^5^ In other words, the progress made in gender equality at the lower grades of academic psychiatry has not had the same rate of impact on the highest grade. This could be due to the fact that people tend to occupy a professorial post for much longer than lower grade posts and thus vacancies do not arise as often. It might therefore be expected to take longer for gender equality to be achieved at this level than at senior lecturer and reader/associate professor level. Nevertheless, 18 years is surely long enough to infer that this is not simply a ‘pipeline’ problem that will correct itself over time.

### London compared with the rest of the UK

We also found that the situation within and outside London differed, with somewhat better gender equality in the capital, where women held 55% of academic psychiatry posts, compared with 28% elsewhere. Outside London, the majority of more junior posts were occupied by men and, although there were equal numbers of men and women at senior lecturer grade and more women than men at reader/associate professor level, there were very few of these posts. At professor level, men held 31 of the 35 available positions. Within London, although research fellow and lecturer posts were in greater supply and women held the majority of these, the *‘pinch point’* in career progression came above senior lecturer level, with women representing only a quarter of all professors. Comparing our results with our 2003 survey of London universities,^7^ we found that the percentage of women at all grades had increased, but statistically significant increases were only evident at lecturer and senior lecturer level. It therefore seems that, although the opportunity for progression in academic psychiatry is improving for women overall across the UK, it remains very challenging to achieve the highest level of promotion, and the situation may be even harder for women pursuing their career outside London.

### Barriers to career progression and the Athena SWAN initiative

The barriers to career progression for women in academia have been described previously,^9^ and helpfully summarised by Howard^10^ as including ‘few visible role models and mentors, the short-term contracts used for relatively senior academic positions, lack of transparency for pay and promotion procedures, gender imbalance in the decision-making processes of promotion and organisational policies, slow setting up and take-up of work life-balance policies and, particularly challengingly, the intangible cultural factors that seem to exclude women from the corridors of power’. Others have also emphasised the importance of unconscious bias as a driver of inequality within academic institutions.^11^

The Athena SWAN charter established guiding principles to assist higher education institutions in addressing the many barriers to gender equality, with the aim of improving the recruitment, retention and career progression of female academics.^8^ Its bronze, silver and gold accreditation awards provide an incentive to establish and develop key actions and policies to overcome the specific barriers in a particular setting, with the aim of changing cultures and processes that disadvantage female staff. The charter has since been extended to non-STEMM specialties and been broadened to include other aspects of diversity as well as gender.

The implementation of the Athena SWAN initiative was evaluated in five departments of one UK medical school using a qualitative approach.^12^ Although it was felt to have introduced a welcome mechanism to raise the issue of gender equality within the organisation, it was also reported to create considerable additional work for female staff. This included completing the lengthy SWAN application itself, as well as coordinating actions to address specific barriers, and the increased burden on the small number of senior women, who had to take on more committee work to improve gender representation. The authors concluded that the aims of the initiative were undermined by the negative impact on female staff.^12^ A separate evaluation found no difference in the career progression of female academics in the 12 UK medical schools that had been participating in the Athena SWAN programme from its inception compared with those that joined after the announcement in 2011 that the award of National Institute of Health Research (NIHR) funding would be contingent on achieving a silver award.^13^

The financial incentivising of the Athena SWAN initiative by the NIHR represents a ‘carrot and stick’ approach which has certainly raised the consciousness of higher education institutions to the pervasive gender inequality they harbour. All the universities we surveyed had joined the Athena SWAN programme and 12 of the 15 held a silver award at the time of our 2019 survey. Our results suggest that in academic psychiatry there has been clear improvement in the representation of women at all grades of academic post since 2001. Nevertheless, even with the support of a national accreditation process and a financial ‘sword of Damocles’ suspended over these institutions, women in academic psychiatry remain disadvantaged in their career progression within them, particularly with regard to the achievement of a professorial post. The ongoing male dominance at the highest academic grade is, put simply, dispiriting.

### Reasons for the continued inequality

One possible reason for this continued disparity is that women are not achieving the key metrics that most universities take into account for promotion to professor. Women tend to hold more teaching and pastoral support roles than their male colleagues, areas of responsibility that tend to be valued less than research activities when considering senior promotions.^14^ As well as research grant income, publications are a common metric for academic promotion. However, no data are available allowing us to compare the academic credentials of applicants for promotion to professor by gender in psychiatry.

Our data raise a further important issue. The Royal College of Psychiatrists’ workforce census of 2001 identified 218 academic psychiatrists at senior lecturer grade and above, but only 153 in the 2019 census. Our survey identified 195 at these grades, of whom 134 were based in London, and in 2003 we identified 165 London-based academic psychiatrists at the same grades. All these data suggest that the number of academic posts in psychiatry is shrinking. Across the UK we identified 248 posts at any grade, representing a total reduction of one-third since 2001. Although this problem is not limited to psychiatry, it seems to have been particularly badly affected. The Medical Schools Council reported a reduction in all senior lecturer and reader posts of between 25 and 33% across the four countries of the UK since 2004 and highlighted that psychiatry had seen major losses – 84.4 full-time equivalent (FTE) senior lecturer/reader posts between 2007 and 2017.^5^ This clearly adds further pressure and challenge for those hoping to enter and progress a career in academic psychiatry.

## Data Availability

The data that support the findings of this study are available on request from the corresponding author.

## About the authors

**Sukriti Dhingra** was an MSc student in Clinical Mental Health Sciences in the Division of Psychiatry, University College London, UK from 2018–2019. **Helen Killaspy** is Professor of Rehabilitation Psychiatry in the Division of Psychiatry, University College London, UK. **Sarah Dowling** is Deputy Manager of the Division of Psychiatry, University College London, UK.
